# Embryo-Endosperm Interaction and Its Agronomic Relevance to Rice Quality

**DOI:** 10.3389/fpls.2020.587641

**Published:** 2020-11-30

**Authors:** Lu An, Yang Tao, Hao Chen, Mingjie He, Feng Xiao, Ganghua Li, Yanfeng Ding, Zhenghui Liu

**Affiliations:** ^1^College of Agriculture, Nanjing Agricultural University, Nanjing, China; ^2^Collaborative Innovation Center for Modern Crop Production, Nanjing Agricultural University, Nanjing, China

**Keywords:** rice, seed development, embryo, endosperm, interaction, grain quality

## Abstract

Embryo-endosperm interaction is the dominant process controlling grain filling, thus being crucial for yield and quality formation of the three most important cereals worldwide, rice, wheat, and maize. Fundamental science of functional genomics has uncovered several key genetic programs for embryo and endosperm development, but the interaction or communication between the two tissues is largely elusive. Further, the significance of this interaction for grain filling remains open. This review starts with the morphological and developmental aspects of rice grain, providing a spatial and temporal context. Then, it offers a comprehensive and integrative view of this intercompartmental interaction, focusing on (i) apoplastic nutrient flow from endosperm to the developing embryo, (ii) dependence of embryo development on endosperm, (iii) regulation of endosperm development by embryo, and (iv) bidirectional dialogues between embryo and endosperm. From perspective of embryo-endosperm interaction, the mechanisms underlying the complex quality traits are explored, with grain chalkiness as an example. The review ends with three open questions with scientific and agronomic importance that should be addressed in the future. Notably, current knowledge and future prospects of this hot research topic are reviewed from a viewpoint of crop physiology, which should be helpful for bridging the knowledge gap between the fundamental plant sciences and the practical technologies.

## Introduction

Rice (*Oryza sativa*) is a model plant for genomic studies, through which the genes discovered provide fundamental insight into the complex and dynamic processes governing plant life. Rice is also one of the most important staple foods worldwide, possessing a tremendous significance not only in Asia but also in Africa and South America (Muthayya et al., 2014). Recently, along with rise in living standard, quality trait like grain chalkiness and eating property has been one of the main objectives for rice breeding and production. However, the molecular and physiological mechanism underlying rice quality is still in its infancy (Fitzgerald et al., 2009; Yang et al., 2013; Li et al., 2018).

**Figure 1:**
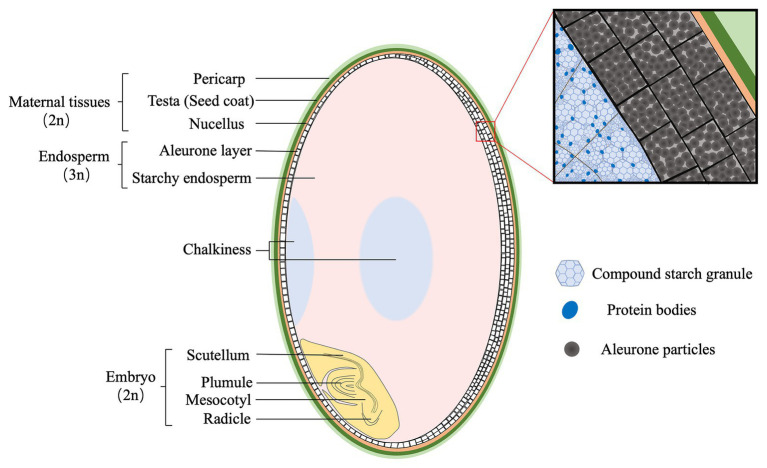
The structure of mature rice grain (caryopsis). The maternal tissues are composed of pericarp (light green), testa (green), and nucellus (yellow), and are located at the outer layer. The endosperm consists of aleurone (white) and starchy endosperm (pink) that are packed with starch granules and protein bodies, respectively. Generally, the aleurone has two or three layers at the dorsal, whereas one at the ventral side of rice grain. Opaque tissues (light blue), called chalkiness, generally occurs at the ventral (white-belly) and central (white-core) parts of endosperm. The embryo (light yellow) is composed of two structures: scutellum and embryonic axis (plumule, mesocotyl, and radicle).

Rice grain (brown rice) consists of three genetically distinctive tissues, the diploid embryo, the triploid endosperm, and the diploid maternal tissues (Figure 1). Embryo is located at the ventral bottom part, and mainly composed of the scutellum and embryonic axis. The scutellum functions as conductive tissue between embryo and endosperm (Wang et al., 2020). Endosperm is composed of two tissues, starchy endosperm and the aleurone. Starchy endosperm is at the center of the grain, while the aleurone occurs in one or more layers surrounding the starchy endosperm and the embryo. The outer layer of rice grain is the supportive and protective maternal tissues, mainly including the pericarp, testa, and nucellus.

Before consumption, brown rice is subjected to milling process to produce milled or polished rice meeting the consumer’s preference (Bao, 2019). Milling has a substantial influence on rice quality and value, by removing the embryo, the maternal tissues, the aleurone, and even part of starchy endosperm depending on the degree of milling. The remaining part of the grain, milled rice, is the starchy endosperm that mainly composed of starch and protein. Therefore, rice quality traits are dependent on the physico-chemical properties of starchy endosperm. For eating quality, it is coordinately controlled by amylose, amylopectin, proteins, and lipids (Bhattacharya, 2011). In addition, the formation of grain chalkiness is attributed to loosely packed starch granules and protein bodies (Xi et al., 2014).

Apparently, the end use quality of rice grain is determined by endosperm properties. However, the composition, structure, and size of the endosperm are actually affected by its interactions with other tissues, in particular the embryo, during the whole process of seed development (Lafon-Placette and Köhler, 2014; Ingram, 2020). Fundamental science of functional genomics has uncovered some key mechanisms regulating embryo and endosperm development, but the interaction or communication between the two tissues is largely elusive. Further, the significance of this interaction for grain filling remains open. This review offers a comprehensive and integrative view of embryo-endosperm interaction, with emphasis on its relevance to rice quality. In particular, current knowledge and future prospects of this hot research topic are reviewed from a viewpoint of crop physiology, which should be helpful for bridging the knowledge gap between the fundamental plant science (gene study) and the practical technology (crop breeding and management).

## Categorizing Developmental Stages of Rice Grain From the Perspective of Agronomy

**Figure 2:**
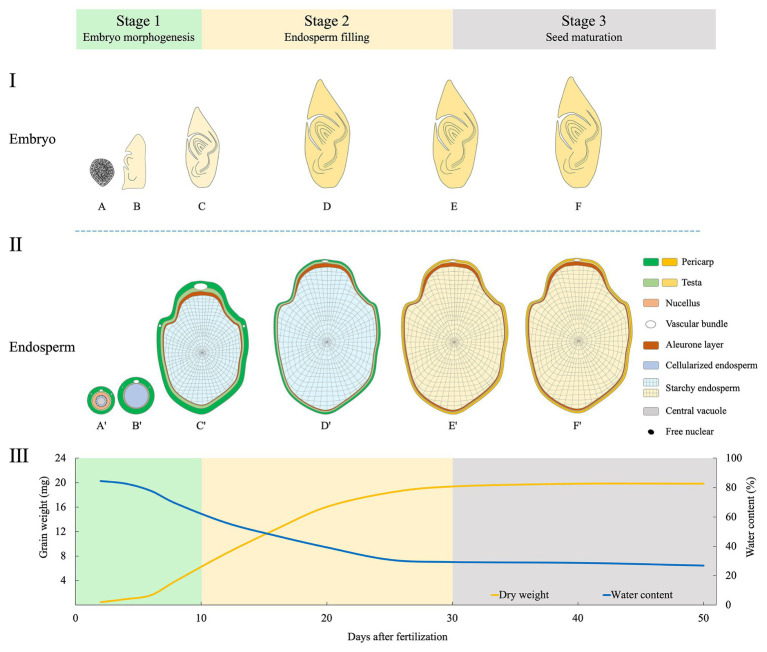
Schematic illustration of morphological changes of embryo (longitudinal section, I), endosperm (transversal, II), and the dynamics of dry matter accumulation (III) during rice grain filling. The three phases proposed are indicated. Changed colors of the pericarp and testa show the process of degradation of maternal tissues (Wu et al., 2016a,b), and that of the starchy endosperm indicates the grain-filling process. The curve of grain weight and water content are depicted by data synthesized from Zhu et al. (2011), Fu et al. (2013), and Wu et al. (2016b). Stage 1 (embryo morphogenesis): At 2 days after fertilization (DAF), embryo is at globular stage **(A)**, and endosperm at the coenocyte stage **(A')**. At 5 DAF, embryo has the first leaf primordium and recognizable scutellum **(B)**, endosperm cellularization is completed **(B')**. At 10 DAF, embryo morphogenesis is basically completed **(C)**; endosperm differentiation is finished, with two structures of the aleurone and starchy endosperm **(C')**. Stage 2 (endosperm filling): During 10–30 DAF, embryo becomes dormant **(D,E)**; endosperm accumulates storage compounds and attains its maximum weight **(D',E')**. Stage 3 (seed maturation): embryo **(F)** and endosperm **(F')** continue to dehydrate until maturity.

Development of rice grain is a delicate and complex process, during which the embryo and endosperm develop synchronously. For fundamental studies, the embryo development has been subdivided into 10 stages based on morphological, anatomical, and molecular landmark events (Itoh et al., 2005). Similarly, rice endosperm development was classified into four stages based on histochemical studies: coenocyte, cellularization, storage product accumulation, and maturation (Wu et al., 2016b). So far, for crop scientists, it is still lacking in a practical staging system that integrates the developmental features of both embryo and endosperm. Here, we propose a new staging system with three phases based on a complete map of the whole grain (Figure 2).

### Stage 1 (0–10 DAF): Embryo Morphogenesis

This stage is dominated by the morphogenesis of the embryo. After fertilization, the embryo undergoes globular stage until 3 days after fertilization (DAF). At 5 DAF, the first leaf primordium is recognizable. From then, enlargement of embryonic organs, especially the scutellum, is remarkable, and this continues until 10 DAF when most morphogenetic events are completed (Itoh et al., 2005). Meanwhile, endosperm undergoes coenocyte stage at 1–2 DAF. Then, the cellularization begins at 3 DAF and finishes the process at 5 DAF. The aleurone emerges at 6 DAF. Small spherical or elliptical amyloplasts are formed at 5 DAF, and protein bodies are initiated at 9 DAF, indicating the starting of storage compound accumulation.

### Stage 2 (10–30 DAF): Endosperm Filling

This stage is characterized by the accumulation of starch and proteins in endosperm. Embryonic organs continue enlargement but at small extent. The embryo matures until 20 DAF and is dormant thereafter. By contrast, grain filling reaches the maximum rate between 10 and 20 DAF (Zhu et al., 2011; Fu et al., 2013). From 21 DAF onward, endosperm enters the maturation stage. Morphologically, aleurone cells show very few changes, while those in starchy endosperm gradually lose their margins. Starch granules are formed in the starchy endosperm when boundaries of single starch granules become less clear (Wu et al., 2016a). There is no dramatic increase in fresh grain weight during 20–30 DAF. Concomitantly, most of the starchy endosperm and maternal tissues, such as vascular bundles, the nucellar projection and the nucellar epidermal cells are degraded at the end of this stage (Wu et al., 2016a,b).

### Stage 3 (30 DAF-Maturity): Seed Maturation

After completion of reserve accumulation, rice seed enters the last period of maturation. This stage is characterized by a major loss of water, culminating in a dry and quiescent seed. Embryo establishes desiccation tolerance and experiences a developmentally programmed dehydration that results in dormancy (Manfre et al., 2009). At maturity, the embryo occupies only a small fraction of the grain; the rest is mostly the starch-rich endosperm. The starchy endosperm cells die upon seed maturation and desiccation, but the tissue retains alive with capacity for some metabolic activities, including a redox system that is crucial for germination (Zeeman, 2015). At this stage, seeds are susceptible to germination under hot and humid conditions, causing preharvest sprouting (PHS) which has detrimental effect on grain yield and quality (Du et al., 2018).

## Interactions Between Embryo and Endosperm During Grain Filling

**Figure 3:**
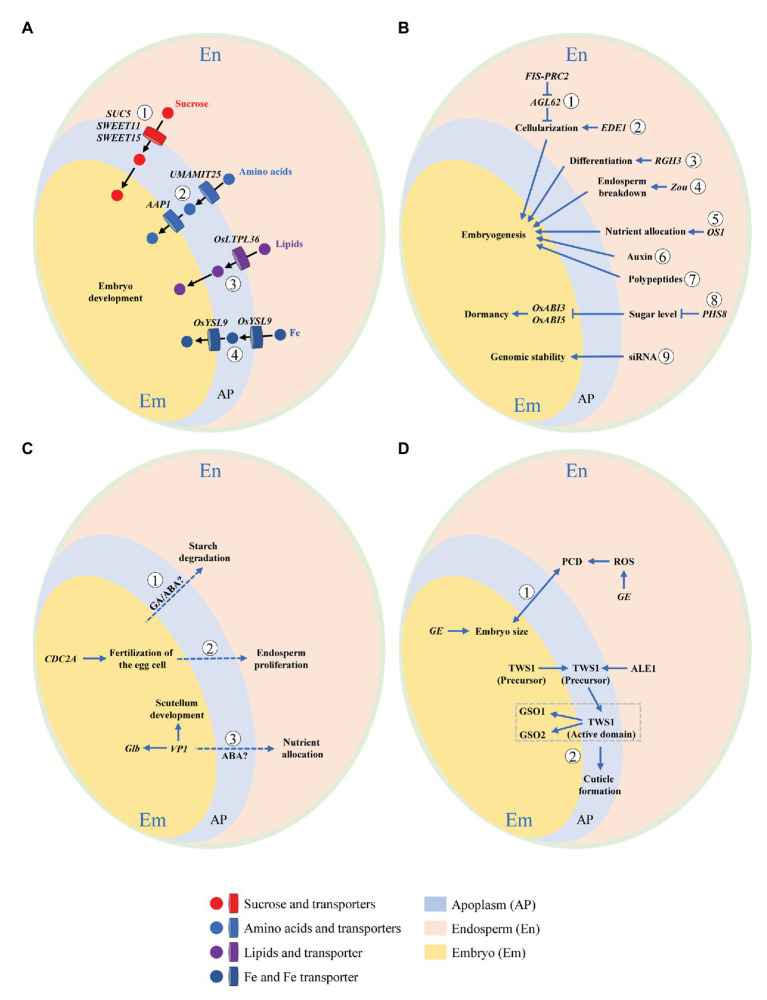
Schematic illustration of embryo-endosperm interactions during rice grain filling. Concomitant development of embryo and endosperm within the limited space of seed coat requires bidirectional dialogues between the two compartments. This interaction can be summarized by the following four aspects. **(A)** Nutrient flow from endosperm to the developing embryo *via* apoplastic transportation. Nutrients and their transporters are indicated. ① Sucrose transporters, *SWEET11*, *SWEET15* (Chen et al., 2015), and *SUC5* (Baud et al., 2005). ② Amino acids transporters, *UMAMIT25* (Besnard et al., 2018), and *AAP1* (Sanders et al., 2009). ③ Lipids transporter, *OsLTPL36* (Wang et al., 2015). ④ Fe transporter, *OsYSL9* (Senoura et al., 2017). **(B)** Dependence of embryo development on endosperm. Genes exclusively expressed in or signaling released from endosperm that regulate embryo development are shown. ① *FIS-PRC2* and *AGL62* (Hehenberger et al., 2012). ② *EDE1* (Pignocchi et al., 2009). ③ *RGH3* (Fouquet et al., 2011). ④ *Zou* (Yang et al., 2008). ⑤ *OS1* (Song et al., 2019). ⑥ Auxin (Chen et al., 2014). ⑦ Polypeptides (Widiez et al., 2017). ⑧ *PHS8* (Du et al., 2018). ⑨ Small-interfering RNA (siRNA; Lafon-Placette and Köhler et al., 2014). **(C)** Regulation of endosperm development by embryo. Genes elusively expressed in or signaling released from embryo that modulate endosperm development are demonstrated. ① GA/ABA (Zeeman, 2015). ② *CDC2A* (Nowack et al., 2006). ③ *VP1* (Zheng et al., 2019). **(D)** Developmental coordination between embryo and endosperm. Bidirectional dialogues are presented by signaling pathways mediated by ① *GE* (Nagasawa et al., 2013) and ② TWS1 (Doll et al., 2020a). Dotted line indicates putative or unproven signaling pathway.

Interaction means a mutual or reciprocal action, or the effect that two things have on each other. On the one hand, endosperm functions as supporting and nurturing the developing embryo, and as such is believed to have a central role in regulating embryo development; but on the other, the embryo is the miniature of next generation, thus being the growth center with a priority of nutrient supply. It is tempting to speculate that some signals be released by the embryo to remobilize endosperm storage when nutrients are not sufficient for supporting its development, as the case of germination. Development of embryo and endosperm is temporally synchronized, thus the two compartments need to be tightly coordinated and interact physically and chemically in order to allow the formation of a viable seed. Understanding these interactions is an interesting but formidable challenge in the drive to clarify mechanisms of resource partitioning among embryo and endosperm, which is critical for the formation of both grain yield (weight) and quality. Growing evidences in rice as well as *Arabidopsis thaliana* and maize (*Zea mays*) support bidirectional communications between the two compartments (Figure 3).

### Nutrient Flow From Endosperm to the Developing Embryo *via* Apoplastic Transportation

At early stage, nutrients for the developing embryo of rice are from two sources, the endosperm and the maternal tissues *via* the nucellus at its base. After 8 DAF, the embryo breaks its connection to the nucellus that has been degraded (Jones and Rost, 1989), and subsequently, endosperm becomes the only source of nutrients. Results from *Arabidopsis* and rice have converged to show that embryo and endosperm become symplastically isolated at or soon after fertilization, indicating that communication between the two compartments must occur across the apoplast (Widiez et al., 2017). The intercompartmental apoplastic interfaces and the cells on each side of them thus represent critical zones for understanding exchanges of nutritional and signaling cues. The flow of nutrients across the apoplastic space depends on transport proteins (transporters) located on the interfaces between endosperm and embryo (Figure 3A). Dysfunction of transporters may result in interruption or retardation of nutrient flow and subsequently arrest of embryo development.

Sucrose, the major form of transporting carbohydrates [or carbon (C)], is transported *via* the maternal tissues to the endosperm, and then is secreted from endosperm to feed embryo by SUTs (for sucrose) and SWEETs (for hexose and sucrose). The sucrose transporter gene *AtSUC5* expression is endosperm specific, being specifically and highly expressed between 4 and 9 DAF. Seeds of *suc5* mutant exhibited a strong but transient reduction in fatty acid content, as was associated with a slight delay in embryo development, demonstrating the role of *AtSUC5* in nurturing endosperm at early developmental stage (Baud et al., 2005). In seed of *Arabidopsis*, three sucrose transporters SWEET 11, 12, and 15 showed specific spatio-temporal expression patterns (Chen et al., 2015). *SWEET11* transcripts were expressed basically in endosperm and seed coat at the stages of linear cotyledon and maturation green. *SWEET12* transcripts accumulated primarily in seed coat at the same two stages, but appeared in suspensor and micropylar at the globular stage. Transcripts of *SWEET15* were detected in endosperm at the globular and maturation green stages. A *sweet11;12;15* triple mutant showed a “wrinkled” seed phenotype with arrested embryo development, suggesting the necessity of this cascade of sequentially expressed SWEETs to nutrient provision to the developing embryo from maternal tissues *via* endosperm.

Phloem-derived amino acids are principal source of nitrogen (N) feeding the developing seeds. Some members of usually multiple acids move in an out transporter (UMAMIT) family have previously been implicated in this process. In *Arabidopsis*, UMAMIT25 is expressed in endosperm cells within developing seeds. Seed amino acid contents of *umamit25* knockout lines were lower during embryogenesis in comparison with the wild type, suggesting that UMAMIT25 might mediate amino acid export from endosperm to the developing embryo (Besnard et al., 2018). AAP1 is an amino acid transporter that was localized to outermost epidermis cells and the parenchyma of *Arabidopsis* embryo (Sanders et al., 2009). In mature and desiccated *aap1* seeds, total N and C content was significantly decreased while total free amino acid content was increased. Separately analyzed embryos and seed coats/endosperm of mature seeds showed that the increase in amino acids was accompanied by an accumulation in the seed coat/endosperm, suggesting that the decreased uptake of amino acids by the *aap1* embryo affects N homeostasis in seed coat and endosperm.

Lipids are mainly stored in embryo and aleurone layer of rice seed. OsLTPL36, a homolog of putative lipid transport protein, is specifically expressed in seed coat and aleurone cells. Suppression of *OsLTPL36* by RNAi caused chalky endosperm and reduced fatty acid content. In addition, the embryo development was delayed with downregulation of *OsLTPL36*. The finding showed that OsLTPL36 performs a role in embryo development (Wang et al., 2015).

The formation of viable seeds in plants requires a continuous unload of minerals from the maternal tissues. In *Arabidopsis*, Zn appears to be reallocated from endosperm at the late globular stage, while Mn at the late bent-cotyledon stage of embryo development (Otegui et al., 2002). In rice, the Fe transporter gene *OsYSL9* was expressed at the interface between embryo and endosperm. In *OsYSL9*-knockdown plants, Fe content was reduced in embryo but elevated in other tissues, suggesting the involvement of *OsYSL9* in intercompartmental Fe homeostasis within rice seed (Senoura et al., 2017).

### Dependence of Embryo Development on Endosperm

Endosperm is the nutrient source to drive embryo growth. It also places significant physical and metabolic constraints on embryo, impeding or even preventing its development entirely. The dependence of embryo development on endosperm is exemplified by a heterofertilization experiment in maize, aimed at empirically testing the notion that genetic relatedness of an endosperm to its compatriot embryo might limit the amount of resources allocated to the embryo (Wu et al., 2013). They found that the degree of genetic relatedness between endosperm and embryo changed the distribution of maternal resources allocated from endosperm to embryo, with lower relatedness coefficient being related with smaller embryo size.

The importance of endosperm cellularization for embryo viability has been proven by mutants deficient in the endosperm-specific *FERTILIZATION INDEPENDENT SEED POLYCOMB COMPLEX2* (*FIS-PRC2*) that has abnormal endosperm cellularization (Figure 3B). Seeds deficient in *FIS-PRC2* function abort, with embryos development arrested at heart stage (Köhler et al., 2003). Embryo arrest can be bypassed by *in vitro* cultivation of dissected embryos, strongly supporting that the failure of endosperm cellularization leads to embryo development retardation (Hehenberger et al., 2012). Similarly, loss function of *ENDOSPERM DEFECTIVE1* (*EDE1*) results in endosperm cellularization failure and embryo retardation at late heart stage (Pignocchi et al., 2009). In addition, the Type I MADS-box transcription factor AGL62, one of the major regulators of endosperm cellularization in *Arabidopsis*, is epigenetically controlled by the FIS-PRC2 complex (Hehenberger et al., 2012).

Endosperm differentiation is controlled by both maternal and paternal genomes (Berger and Chaudhury, 2009). *Rough Endosperm 3* (*RGH3*), encoding a predicted RNA splicing factor, is required for a subset of RNA splicing event during maize seed development (Fouquet et al., 2011). In the *rgh3* mutant, the embryo surrounding region (ESR) and basal endosperm transfer layer (BETL) cell differentiation is defective, and mature *rgh3* seeds have a rough and pitted endosperm surface, and show different degrees of embryo defects. The defective phenotype of endosperm and embryo suggests *rgh3* endosperm negatively regulates embryo development. In addition, it indicates that alternative RNA splicing mediated by *Rgh3* could be essential for endosperm differentiation and embryo development (Fouquet et al., 2011).

Endosperm breakdown is fundamental for embryo growth, thereby physically providing space and remobilizing its stored nutrients to drive embryo development. A basic helix–loop–helix (bHLH) transcription factor, ZHOUPI (ZOU) mediates the processes of endosperm degradation and nutrient remobilization in *Arabidopsis*. ZOU is expressed exclusively around the ESR in the endosperm of developing seeds (Ingram, 2010). The *zou* mutant retains more endosperm cells than do wild-type at maturity, and its embryo has defects in cuticle formation and in epidermal cell adhesion, indicating that ZOU functions non-autonomously to control endosperm breakdown and regulate embryonic development (Yang et al., 2008). In maize, ZmZOU also has a similar active role in ESR and/or suspensor degradation during embryo development (Grimault et al., 2015).

In maize, the BETL is crucial for uptake and allocation of nutrients from maternal tissues to filial tissues of endosperm and embryo. *OPAQUE ENDOSPERM AND SMALL GERM 1*(*OS1*) encodes a putative transcription factor with an RWP-RK domain (Song et al., 2019). It is expressed especially in BETL, conducting zone, and central starch endosperm cells. The *os1* mutation causes opacity at the endosperm crown, and meanwhile a smaller embryo. RNA sequencing demonstrated that many genes involved in nutrient allocation and storage are significantly downregulated in the *os1* mutant, suggesting that *OS1* plays a role in nutrient allocation between embryo and endosperm.

The phytohormone auxin is a key regulator required for seed development. Auxin signaling from endosperm regulates the development of embryo. A recent study of hormonal responses in the maize kernel highlighted an interesting potential role of ESR in preventing auxin fluxes from endosperm to embryo at very early developmental stages (Chen et al., 2014). Interestingly, Chen et al. (2014) observed elevated auxin signaling activity in the embryo-surrounding endosperm above ESR, which was correlated with the activation of basally directed auxin fluxes and auxin responses in the apical regions of embryo once it differentiated from ESR. Based on these findings, it was proposed that endosperm derived auxin could be critically important in early embryo patterning in maize.

Additionally, there are other novel signaling pathways involved in embryo-endosperm communication, such as sugar, small-interfering RNA (siRNA), and polypeptides. Trehalose-6-phosphate (T6P), a dominant sugar signal in plants, modulates sucrose metabolism and allocation, thus performing an active role in regulating crop growth and development (Griffiths et al., 2016). In wheat grain, T6P sucrose was at high levels in endosperm but was low in pericarp and embryo, indicating a tissue-specific regulation of sucrose metabolism by T6P (Martínez-Barajas et al., 2011). *PHS8* encodes a starch-debranching enzyme, isoamylase 1 (ISA1). Mutation in *PHS8* causes the phytoglycogen breakdown and sugar accumulation in endosperm, which is accompanied by preharvest sprouting of the matured embryo. This result suggests that sugar in endosperm not only acts as a fundamental energy source but also as a signal mediating embryo development (Du et al., 2018). The role of siRNA and polypeptides in intercompartmental coordination in developing seed was in depth reviewed by Lafon-Placette and Köhler (2014) and Widiez et al. (2017).

### Regulation of Endosperm Development by Embryo

Nutrients unloaded from the maternal tissues are first stored in endosperm. Then, they are either utilized by endosperm to synthesize storage compounds like starch and proteins, or transferred to the developing embryo. In this regard, endosperm dominates nutrient allocation among the compartments within a seed. However, it is the embryo that locates at the center of seed development, and thus, the role of endosperm is widely accepted as being altruistic, ready to “sacrifice” itself to nurture the next generation, the embryo (Wu et al., 2013; Ingram, 2020). A tempting speculation is, therefore, that embryo exerts a “counter-acting regulation force” on endosperm, as supported by mounting evidences.

**Figure 4:**
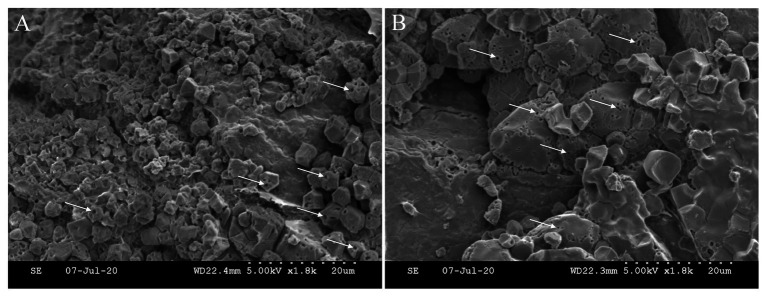
Starch degradation in the endosperm cells facing the scutellum of the embryo. Micro-pores or pits (white arrow) on the surface of starch granule indicate the hydrolysis of starch reserve by α-amylase. The location of **(A)** is nearer to embryo (scutellum) than that of **(B)**. Note that starch granules are smaller at location **(A)** than those at **(B)**. The immature starch granules at location **(A)** might be the result of nutrient deprivation by the developing embryo.

The direct evidence of the impact of embryo on endosperm development may come from the scanning electron microscopy (SEM) photos that show the decomposition of starch granules in the endosperm cells facing the scutellum of embryo (Figure 4). Micro-pores or pits are highly visible on the surface of starch granules, confirming hydrolysis of starch reserve by α-amylase (Lin et al., 2016; Nakata et al., 2017). In addition, these pits mainly occur at the interfaces between the two compartments, thus it is tempting to assume that the degradation of starch is associated with the urgent demanding for sugars by embryonic development. Whether signaling of GA from embryo plays a role in decomposing endosperm starch remains unclear.

The signal released from embryo regulates endosperm development (Figure 3C). Nowack et al. (2006) characterized a mutation of the *Arabidopsis* Cdc2 homolog *CDC2A*, of which the mutant pollen can fertilize only one cell, instead of two, in the embryo sac, thus making it possible for a genetic dissection of the double fertilization process. They found that upon exclusive fertilization of the egg cell, the unfertilized endosperm also began to develop, suggesting a positive signal from the fertilized egg cell (the embryo) to the central cell (the endosperm) at early stage. Growing evidence in maize and *Arabidopsis* suggest that ESR involves in nurturing and defensing the embryo, and also mediate signaling between embryo and endosperm (Widiez et al., 2017). Recently, a newly interface tissue, named endosperm adjacent to scutellum (EAS), was identified by the analysis of genome-wide expression profiles of tissues at the interfaces between embryo and endosperm in maize seed. The EAS appears around 9 DAF and persists for around 11 days, with enrichment transcriptome of genes encoding transporters. The absence of embryo in an embryo specific mutant can change the expression pattern of the marker genes, suggesting a role of embryo in controlling this specific endosperm tissue (Doll et al., 2020b).

The embryo also regulates nutrient allocation between itself and endosperm. *VIVIPAROUS 1* (*VP1*), is a B3 family TF participating in abscisic acid (ABA) mediated transcriptional regulation (McCarty et al., 1991). Loss of *VP1* function leads to preharvest sprouting in maize, showing a viviparous phenotype. *VP1* starts to be expressed in embryo at 5 DAF, particularly in scutellum (the nutrient transfer link between embryo and endosperm), possibly functioning as mediator for their interactions (Cao et al., 2007; Meng et al., 2018). Recent study suggests that *VP1* senses the nutritional status of endosperm through endosperm-embryo communication, likely the ABA signaling pathway. It regulates scutellum development, enlarges embryo nutrient-storage capacity, and consequently mediates nutrient remobilization from endosperm to embryo (Zheng et al., 2019).

### Developmental Coordination Between Embryo and Endosperm

One of the evolutive advantages of double fertilization in angiosperms is the coordinating development of endosperm and embryo that minimizes nutrient and energy wastage (Ingram, 2020). Belmonte et al. (2013) presented a global gene expression profiles in regions and subregions of the *Arabidopsis* seed from fertilization through maturity. They identified strong overlap in embryo and endosperm expression programs, suggesting a substantial coordination of embryo and endosperm in terms of biological processes. Further, the results were in favor of the embryo-based evolutionary origin of the endosperm, although not excluding the possibility that homology may exist between endosperm and the female gametophyte.

Concomitant development of the two compartments within the confined space of seed coat demands bidirectional dialogues to allow successful seed development and to establish a new competent sporophytic generation (Lafon-Placette and Köhler, 2014; Figure 3D). This was verified by a set of mutants with contrasting embryo/endosperm size balance (Zhou et al., 2013). The rice *GIANT EMBRYO* (*GE*) gene encoding CYP78A13 is essential for controlling size balance of embryo and endosperm (Nagasawa et al., 2013). It is strongly expressed in the interfacing tissues between embryo and endosperm. Heterofertilization and transgenic experiments proved that *GE* performs its function in both embryo and endosperm to acquire a delicate size balance between them, which is associated with the regulation of ROS homeostasis and cell death in endosperm. Some signals responsible for the size balance may be originated from endosperm to embryo and vice versa. In addition, considering space limitation by the glumes (lemma and palea), it is likely that cell death is the solution to embryo-endosperm conflict (Nagasawa et al., 2013).

The bidirectional dialogues between embryo and endosperm have been clearly proven by a recent work on a sulfated peptide, TWISTED SEED1 (TWS1). A hydrophobic cuticle around the embryo prevents it from catastrophic dehydration at early germinating stage. The formation of embryonic cuticle is regulated by the ABNORMAL LEAF SHAPE1 subtilase and the two GASSHO receptor-like kinases, GSO1 and GSO2. Doll et al. (2020a) identified a back-and-forth signaling pathway responsible for the integrity of embryonic cuticle. The TWS1 precursor is produced in embryo and then translocated to endosperm, where it is transformed into an active form. The active peptide shuttles back into the embryo to act with the receptors of GSO1 and GSO2, thus activating local gap repair. The shuttle of TWS1 continues until all gaps are bridged to form an intact cuticle.

## Relevance of Embryo-Endosperm Interaction to Rice Quality: Grain Chalkiness as an Example

Physically and chemically, rice quality refers to the properties of starchy endosperm packed with starch granules and protein bodies. And most of the studies on quality traits had been centered on endosperm, and concluded that C and N metabolisms and their interaction form the foundation for traits like appearance (chalkiness) and eating quality (Xi et al., 2014; Lin et al., 2017; Wada et al., 2019). However, the role of embryo needs to be thoroughly estimated, due to its substantial impact on endosperm development and storage accumulation, as discussed above.

Chalkiness is the opaque tissue feature that usually occurs on the ventral (white-belly) and central (white-core) parts of rice endosperm (Figure 1). It is a highly undesirable trait that negatively affects not only appearance quality but also milling, eating, and cooking quality. High occurrence of chalky grains has been a major obstacle for rice producers, especially under the scenario of global warming (Jagadish et al., 2015). The physiological and molecular mechanisms underlying chalkiness formation remain largely elusive, because of the complex interactions between the regulating genes and the environmental factors.

Lin et al. (2014) identified a notched-belly mutant with a high percentage of white-belly that only occurs in the base part of endosperm near the embryo, with the upper half part being translucent. Using this mutant, they developed a novel comparison system that can clarify the mechanisms underlying grain chalkiness. Notably, the comparison is conducted within the same grain, thus forming a nearly identical genetic background to minimize the compound effects of genetic background and growing environment. Employing this comparison system, Lin et al. (2017) performed a complementary proteome and transcriptome profiling of gene activity in the developing grains of the mutant. Some novel key pathways were highlighted as being involved in chalky tissue formation, especially the interaction between C and N metabolisms, the downregulation of ribosomal proteins, and the decreased accumulation of 13 kDa prolamin subunit. More importantly, substantial influence of the embryo on endosperm composition was revealed, with the embryo negatively affecting the storage of total protein, amino acids, and minerals in the chalky endosperm (Lin et al., 2016). Further, this was associated with the upregulation of genes responsible for the transporters for metabolites and the signal messengers of hormones in the chalky endosperm, and therefore, embryo may be involved in regulating the nutrient distribution within the seed (Lin et al., 2017).

Another example showing the potential role of embryo in chalkiness formation is the induction of α-amylase in endosperm by high temperature (Yamakawa and Hakata, 2010; Nakata et al., 2017). Results of Hakata et al. (2012) showed heat stress induced the expression of α-amylase genes, including *Amy1A*, *Amy1C*, *Amy3A*, *Amy3D*, and *Amy3E*, as well as the activity of α-amylase, but lowered the content of an α-amylase-repressing plant hormone, ABA. Further, RNAi-mediated suppression of α-amylase genes produced rice plant with fewer chalky grains under high temperature, supporting that activation of α-amylase is a crucial trigger for chalky tissue formation under heat stress. It is well-established that upon germination, the release of GA from the embryo triggers the secretion of starch-degrading enzymes like α-amylase from the aleurone and the scutellum (Zeeman, 2015). However, it is still unclear whether there exists a similar pathway in developing seed of cereals.

In *Arabidopsis*, endosperm undergoes programmed cell death (PCD) and is decomposed after completing its nourishment function. The induction of α-amylase (*AMY1*) by GA is tightly correlated with nutrient remobilization and the commence of PCD at early stage (Sreenivasulu et al., 2010). On the other hand, in barley (*Hordeum vulgare*) that has a persistent endosperm, genes encoding hydrolases were not expressed during endosperm PCD in the maturing seed, as explained by the expression of α-amylase inhibitors (Sreenivasulu et al., 2010). Accordingly, Sreenivasulu and Wobus (2013) proposed that the GA-α-amylase-PCD network may be equally activated during seed development in a highly time-specific manner for different species. In developing rice grain, especially under heat stress, part of the endosperm starch granule decomposes. However, the signaling pathway responsible for it is largely unknown. In addition, in the mutant of *PHS8/ISA1* with a phenotype of preharvest sprouting, isoamylase was activated, as explained by the suppression of *OsABI3* and *OsABI5*, and thus the reduced sensitivity to ABA (Du et al., 2018). Intriguingly, the involvement of GA in regulating starch hydrolysis was excluded. Similarly, Hakata et al. (2012) argued that the activation of α-amylase under heat stress might to be associated with removal of ABA, but not with the induction of GA. Overall, these results imply divergent hormonal signals modulating starch degradation between developing and germinating stages for cereal grains.

## Summary and Perspectives

Recent advances in *Arabidopsis* and maize have conformed substantial bidirectional interactions between embryo and endosperm during seed development, indicating that the interplay of the two compartments should be common among plant species. As a monocotyledon plant, rice seed is structurally different with that of the dicotyledon *Arabidopsis*. And it is also appreciably different with the maize, not containing conductive endosperm tissues like BETL and ESR. The direct relevance of data from *Arabidopsis* and maize for rice therefore remains unclear. Several key questions need to be answered.

- Signaling controlling the developmental transition of the endosperm. The developmental transitions in endosperm like cellularization of the syncytium are dramatic and abrupt, meaning that gene expression patterns are globally and rapidly reprogrammed (Sabelli and Larkins, 2009). Mechanisms underlying the duration of syncytial phase of endosperm have captured attention of crop scientists, for there exists a positive relation between duration of this phase and grain size and yield (Ishimaru et al., 2013; Olsen, 2020). What signals trigger the major endosperm developmental transitions remains unknown. Does this signaling pathway originate from the embryo that undergoes morphogenesis?
- Hormonal regulation of starch degradation. Much knowledge about starch degradation has been derived from biochemical analyses on germinating cereal grain, especially barley and wheat (*Triticum aestivum*). During germination, GA signaling from embryo triggers the secretion of starch-degrading enzymes from the living aleurone layer and scutellum (Zeeman, 2015). It is still uncertain whether the hydrolyzation of endosperm starch during grain filling is analogous to that of the germination. Do these two processes have a mirror-image relationship? What is the role of GA in regulating starch degradation in developing endosperm? Is there a balance between starch biosynthesis and degradation as controlled by the GA/ABA ratio? What is the relevance of this signaling to chalky tissue formation? And what is the function of aleurone layer in embryo-endosperm interaction?
- Cellular events at late stage. Accumulating literature indicates that the duration of embryo morphogenesis and endosperm filling would be conserved, with the former ending at 10 DAF while the latter at 30 DAF (Itoh et al., 2005; Zhu et al., 2011; Fu et al., 2013; Wu et al., 2016b). After that, the seed enters the stage of desiccation and maturation, lasting for 20–40 days with no obvious increase in grain weight. There is scarcer information concerning the cellular events at the late stage. If that developmental pattern is common among rice cultivars, is there an embryo-endosperm interaction at the late stage of grain filling? And what does this mean to preharvest sprouting?

All the above questions ultimately relate to the physiological and molecular controls of the intercompartmental coordination between embryo and endosperm, which is largely elusive and should be an important future focus. Currently, there exists a knowledge gap between the fundamental plant sciences and the applied technology of crop breeding and management, as partially reflected by the limited progress in high-yielding and quality breeding in sharp contrast to the quantum leap in rice functional genomics. This review calls for cooperative endeavors between crop geneticists, breeders, physiologists, and agronomists to address the crucial events implied in the embryo-endosperm interaction, especially revisiting the role of embryo development in grain filling and rethinking the strategies involved in the high yielding and high quality rice crops.

## Author Contributions

ZL conceived, designed, and wrote the manuscript. LA and YT made the figures. HC, FX, MH, GL, and YD revised the manuscript. All authors contributed to the article and approved the submitted version.

### Conflict of Interest

The authors declare that the research was conducted in the absence of any commercial or financial relationships that could be construed as a potential conflict of interest.

## References

[ref1] Bao J. S. (2019). “Rice milling quality” in Rice. 4th Edn. (Duxford: Elsevier), 339–369.

[ref2] Baud S. Wuillème S. Lemoine R. Kronenberger J. Caboche M. Lepiniec L.. (2005). The AtSUC5 sucrose transporter specifically expressed in the endosperm is involved in early seed development in *Arabidopsis*. Plant J. 43, 824–836. 10.1111/j.1365-313X.2005.02496.x, PMID: 16146522

[ref3] Belmonte M. F. Kirkbride R. C. Stone S. L. Pelletier J. M. Bui A. Q. Yeung E. C.. (2013). Comprehensive developmental profiles of gene activity in regions and subregions of the *Arabidopsis* seed. Proc. Natl. Acad. Sci. U. S. A. 110, E435–E444. 10.1073/pnas.1222061110, PMID: 23319655 PMC3562769

[ref4] Berger F. Chaudhury A. (2009). Parental memories shape seeds. Trends Plant Sci. 14, 550–556. 10.1016/j.tplants.2009.08.003 19748816

[ref5] Besnard J. Zhao C. S. Avice J. C. Vitha S. Hyodo A. Pilot G.. (2018). *Arabidopsis* UMAMIT24 and 25 are amino acid exporters involved in seed loading. J. Exp. Bot. 69, 5221–5232. 10.1093/jxb/ery302, PMID: 30312461 PMC6184519

[ref6] Bhattacharya K. R. (ed.) (2011). “Eating quality of rice” in Rice quality. (Cambridge: Woodhead Publishing), 193–246.

[ref7] Cao X. Y. Costa L. M. Biderre-Petit C. Kbhaya B. Dey N. Perez P.. (2007). Abscisic acid and stress signals induce *Viviparous1* expression in seed and vegetative tissues of maize. Plant Physiol. 143, 720–731. 10.1104/pp.106.091454, PMID: 17208960 PMC1803740

[ref8] Chen J. Y. Lausser A. Dresselhaus T. (2014). Hormonal responses during early embryogenesis in maize. Biochem. Soc. Trans. 42, 325–331. 10.1042/BST20130260, PMID: 24646239

[ref9] Chen L. Q. Lin I. W. Qu X. Q. Sosso D. McFarlane H. E. Londoño A.. (2015). A cascade of sequentially expressed sucrose transporters in the seed coat and endosperm provides nutrition for the *Arabidopsis* embryo. Plant Cell 27, 607–619. 10.1105/tpc.114.134585, PMID: 25794936 PMC4558658

[ref10] Doll N. M. Just J. Brunaud V. Caïus J. Grimault A. Depège-Fargeix N.. (2020b). Transcriptomics at maize embryo/endosperm interfaces identifies a transcriptionally distinct endosperm subdomain adjacent to the embryo scutellum. Plant Cell 32, 833–852. 10.1105/tpc.19.00756, PMID: 32086366 PMC7145466

[ref11] Doll N. M. Royek S. Fujita S. Okuda S. Chamot S. Stintzi A.. (2020a). A two-way molecular dialogue between embryo and endosperm is required for seed development. Science 367, 431–435. 10.1126/science.aaz4131, PMID: 31974252

[ref12] Du L. Xu F. Fang J. Gao S. P. Tang J. Y. Fang S.. (2018). Endosperm sugar accumulation caused by mutation of *PHS8/ISA1* leads to pre-harvest sprouting in rice. Plant J. 95, 545–556. 10.1111/tpj.13970, PMID: 29775500

[ref13] Fitzgerald M. A. McCouch S. R. Hall R. D. (2009). Not just a grain of rice: the quest for quality. Trends Plant Sci. 14, 133–139. 10.1016/j.tplants.2008.12.004, PMID: 19230745

[ref14] Fouquet R. Martin F. Fajardo D. S. Gault C. M. Gómez E. Tseung C. W.. (2011). *Maize Rough Endosperm3* encodes an RNA splicing factor required for endosperm cell differentiation and has a nonautonomous effect on embryo development. Plant Cell 23, 4280–4297. 10.1105/tpc.111.092163, PMID: 22138152 PMC3269866

[ref15] Fu J. Xu Y. J. Chen L. Yuan L. M. Wang Z. Q. Yang J. C. (2013). Changes in enzyme activities involved in starch synthesis and hormone concentrations in superior and inferior spikelets and their association with grain filling of super rice. Rice Sci. 20, 120–128. 10.1016/S1672-6308(13)60116-X

[ref16] Griffiths C. A. Sagar R. Geng Y. Q. Primavesi L. F. Patel M. K. Passarelli M. K.. (2016). Chemical intervention in plant sugar signalling increases yield and resilience. Nature 540, 574–578. 10.1038/nature20591, PMID: 27974806

[ref17] Grimault A. Gendrot G. Chamot S. Widiez T. Rabillé H. Gérentes M. F.. (2015). ZmZHOUPI, an endosperm-specific basic helix-loop-helix transcription factor involved in maize seed development. Plant J. 84, 574–586. 10.1111/tpj.13024, PMID: 26361885

[ref18] Hakata M. Kuroda M. Miyashita T. Yamaguchi T. Kojima M. Sakakibara H.. (2012). Suppression of α-amylase genes improves quality of rice grain ripened under high temperature. Plant Biotechnol. J. 10, 1110–1117. 10.1111/j.1467-7652.2012.00741.x, PMID: 22967050

[ref19] Hehenberger E. Kradolfer D. Köhler C. (2012). Endosperm cellularization defines an important developmental transition for embryo development. Development 139, 2031–2039. 10.1242/dev.077057, PMID: 22535409

[ref20] Ingram G. C. (2010). Family life at close quarters: communication and constraint in angiosperm seed development. Protoplasma 247, 195–214. 10.1007/s00709-010-0184-y, PMID: 20661606

[ref21] Ingram G. C. (2020). Family plot: the impact of the endosperm and other extra-embryonic seed tissues on angiosperm zygotic embryogenesis. F1000Research 9:F1000. 10.12688/f1000research.21527.1, PMID: 32055398 PMC6961419

[ref22] Ishimaru K. Hirotsu N. Madoka Y. Murakami N. Hara N. Onodera H.. (2013). Loss of function of the IAA-glucose hydrolase gene *TGW6* enhances rice grain weight and increases yield. Nat. Genet. 45, 707–711. 10.1038/ng.2612, PMID: 23583977

[ref23] Itoh J. I. Nonomura K. I. Ikeda K. Yamaki S. Inukai Y. Yamagishi H.. (2005). Rice plant development: from zygote to spikelet. Plant Cell Physiol. 46, 23–47. 10.1093/pcp/pci501, PMID: 15659435

[ref24] Jagadish S. V. K. Murty M. V. R. Quick W. P. (2015). Rice responses to rising temperatures-challenges, perspectives and future directions. Plant Cell Environ. 38, 1686–1698. 10.1111/pce.12430, PMID: 25142172

[ref25] Jones T. J. Rost T. L. (1989). Histochemistry and ultrastructure of rice (*Oryza sativa*) zygotic embryogenesis. Am. J. Bot. 76, 504–520. 10.2307/2444345

[ref26] Köhler C. Hennig L. Bouveret R. Gheyselinck J. Grossniklaus U. Gruissem W. (2003). *Arabidopsis* MSI1 is a component of the MEA/FIE *Polycomb* group complex and required for seed development. EMBO J. 22, 4804–4814. 10.1093/emboj/cdg444, PMID: 12970192 PMC212713

[ref27] Lafon-Placette C. Köhler C. (2014). Embryo and endosperm, partners in seed development. Curr. Opin. Plant Biol. 17, 64–69. 10.1016/j.pbi.2013.11.008, PMID: 24507496

[ref28] Li Y. Xiao J. H. Chen L. L. Huang X. H. Cheng Z. K. Han B.. (2018). Rice functional genomics research: past decade and future. Mol. Plant 11, 359–380. 10.1016/j.molp.2018.01.007, PMID: 29409893

[ref29] Lin Z. M. Wang Z. X. Zhang X. C. Liu Z. H. Li G. H. Wang S. H.. (2017). Complementary proteome and transcriptome profiling in developing grains of a notched-belly rice mutant reveals key pathways involved in chalkiness formation. Plant Cell Physiol. 58, 560–573. 10.1093/pcp/pcx001, PMID: 28158863 PMC5444571

[ref30] Lin Z. M. Zhang X. C. Yang X. Y. Li G. H. Tang S. Wang S. H.. (2014). Proteomic analysis of proteins related to rice grain chalkiness using iTRAQ and a novel comparison system based on a notched-belly mutant with white-belly. BMC Plant Biol. 14:163. 10.1186/1471-2229-14-163, PMID: 24924297 PMC4072481

[ref31] Lin Z. M. Zheng D. Y. Zhang X. C. Wang Z. X. Lei J. C. Liu Z. H.. (2016). Chalky part differs in chemical composition from translucent part of japonica rice grains as revealed by a notched-belly mutant with white-belly. J. Sci. Food Agric. 96, 3937–3943. 10.1002/jsfa.7793, PMID: 27166835 PMC5089642

[ref32] Manfre A. J. LaHatte G. A. Climer C. R. Marcotte W. R. (2009). Seed dehydration and the establishment of desiccation tolerance during seed maturation is altered in the *Arabidopsis thaliana* mutant *atem6-1*. Plant Cell Physiol. 50, 243–253. 10.1093/pcp/pcn185, PMID: 19073649

[ref33] Martínez-Barajas E. Delatte T. Schluepmann H. de Jong G. J. Somsen G. W. Nunes C.. (2011). Wheat grain development is characterized by remarkable trehalose 6-phosphate accumulation pregrain filling: tissue distribution and relationship to SNF1-related protein kinase1 activity. Plant Physiol. 156, 373–381. 10.1104/pp.111.174524, PMID: 21402798 PMC3091070

[ref34] McCarty D. R. Hattori T. Carson C. B. Vasil V. Lazar M. Vasil I. K. (1991). The *Viviparous-1* developmental gene of maize encodes a novel transcriptional activator. Cell 66, 895–905. 10.1016/0092-8674(91)90436-3, PMID: 1889090

[ref35] Meng D. X. Zhao J. Y. Zhao C. Luo H. S. Xie M. J. Liu R. Y.. (2018). Sequential gene activation and gene imprinting during early embryo development in maize. Plant J. 93, 445–459. 10.1111/tpj.13786, PMID: 29172230

[ref36] Muthayya S. Sugimoto J. D. Montgomery S. Maberly G. F. (2014). An overview of global rice production, supply, trade, and consumption. Ann. N. Y. Acad. Sci. 1324, 7–14. 10.1111/nyas.12540, PMID: 25224455

[ref37] Nagasawa N. Hibara K. I. Heppard E. P. Vander Velden K. A. Luck S. Beatty M.. (2013). GIANT EMBRYO encodes CYP78A13, required for proper size balance between embryo and endosperm in rice. Plant J. 75, 592–605. 10.1111/tpj.12223, PMID: 23621326

[ref38] Nakata M. Fukamatsu Y. Miyashita T. Hakata M. Kimura R. Nakata Y.. (2017). High temperature-induced expression of rice α-amylases in developing endosperm produces chalky grains. Front. Plant Sci. 8:2089. 10.3389/fpls.2017.02089, PMID: 29270189 PMC5723670

[ref39] Nowack M. K. Grini P. E. Jakoby M. J. Lafos M. Koncz C. Schnittger A. (2006). A positive signal from the fertilization of the egg cell sets off endosperm proliferation in angiosperm embryogenesis. Nat. Genet. 38, 63–67. 10.1038/ng1694, PMID: 16311592

[ref40] Olsen O. A. (2020). The modular control of cereal endosperm development. Trends Plant Sci. 25, 279–290. 10.1016/j.tplants.2019.12.003, PMID: 31956036

[ref41] Otegui M. S. Capp R. Staehelin L. A. (2002). Developing seeds of *Arabidopsis* store different minerals in two types of vacuoles and in the endoplasmic reticulum. Plant Cell 14, 1311–1327. 10.1105/tpc.010486, PMID: 12084829 PMC150782

[ref42] Pignocchi C. Minns G. E. Nesi N. Koumproglou R. Kitsios G. Benning C.. (2009). ENDOSPERM DEFECTIVE1 is a novel microtubule-associated protein essential for seed development in *Arabidopsis*. Plant Cell 21, 90–105. 10.1105/tpc.108.061812, PMID: 19151224 PMC2648083

[ref43] Sabelli P. A. Larkins B. A. (2009). The development of endosperm in grasses. Plant Physiol. 149, 14–26. 10.1104/pp.108.129437, PMID: 19126691 PMC2613697

[ref44] Sanders A. Collier R. Trethewy A. Gould G. Sieker R. Tegeder M. (2009). AAP1 regulates import of amino acids into developing *Arabidopsis* embryos. Plant J. 59, 540–552. 10.1111/j.1365-313X.2009.03890.x, PMID: 19392706

[ref45] Senoura T. Sakashita E. Kobayashi T. Takahashi M. Aung M. S. Masuda H.. (2017). The iron-chelate transporter OsYSL9 plays a role in iron distribution in developing rice grains. Plant Mol. Biol. 95, 375–387. 10.1007/s11103-017-0656-y, PMID: 28871478

[ref46] Song W. B. Zhu J. J. Zhao H. M. Li Y. N. Liu J. T. Zhang X. B.. (2019). OS1 functions in the allocation of nutrients between the endosperm and embryo in maize seeds. J. Integr. Plant Biol. 61, 706–727. 10.1111/jipb.12755, PMID: 30506638

[ref47] Sreenivasulu N. Borisjuk L. Junker B. H. Mock H. P. Rolletschek H. Seiffert U. (2010). “Barley grain development: toward an integrative view” in International review of cell and molecular biology. ed. Jeon K. W. (San Diego: Elsevier Academic Press Inc), 49–89. 10.1016/S1937-6448(10)81002-0 20460183

[ref48] Sreenivasulu N. Wobus U. (2013). Seed-development programs: a systems biology-based comparison between dicots and monocots. Annu. Rev. Plant Biol. 64, 189–217. 10.1146/annurev-arplant-050312-120215, PMID: 23451786

[ref49] Wada H. Hatakeyama Y. Onda Y. Nonami H. Nakashima T. Erra-Balsells R.. (2019). Multiple strategies for heat adaptation to prevent chalkiness in the rice endosperm. J. Exp. Bot. 70, 1299–1311. 10.1093/jxb/ery427, PMID: 30508115 PMC6382329

[ref50] Wang H. T. Zhang Y. M. Xiao N. Zhang G. Wang F. Chen X. Y.. (2020). Rice GERMIN-LIKE PROTEIN 2-1 functions in seed dormancy under the control of abscisic acid and gibberellic acid signaling pathways. Plant Physiol. 183, 1157–1170. 10.1104/pp.20.00253, PMID: 32321839 PMC7333727

[ref51] Wang X. Zhou W. Lu Z. H. Ouyang Y. D. O C. S. Yao J. L. (2015). A lipid transfer protein, OsLTPL36, is essential for seed development and seed quality in rice. Plant Sci. 239, 200–208. 10.1016/j.plantsci.2015.07.016, PMID: 26398804

[ref52] Widiez T. Ingram G. C. Gutiérrez-Marcos J. F. (2017). “Embryo-endosperm-sporophyte interactions in maize seeds” in Maize kernel development. ed. Larkins B. A. (Wallingford: CAB International), 95–107.

[ref53] Wu C. C. Diggle P. K. Friedman W. E. (2013). Kin recognition within a seed and the effect of genetic relatedness of an endosperm to its compatriot embryo on maize seed development. Proc. Natl. Acad. Sci. U. S. A. 110, 2217–2222. 10.1073/pnas.1220885110, PMID: 23345441 PMC3568322

[ref54] Wu X. B. Liu J. X. Li D. Q. Liu C. M. (2016a). Rice caryopsis development I: dynamic changes in different cell layers. J. Integr. Plant Biol. 58, 772–785. 10.1111/jipb.12440, PMID: 26472484 PMC5064628

[ref55] Wu X. B. Liu J. X. Li D. Q. Liu C. M. (2016b). Rice caryopsis development II: dynamic changes in the endosperm. J. Integr. Plant Biol. 58, 786–798. 10.1111/jipb.12488, PMID: 27449987

[ref56] Xi M. Lin Z. M. Zhang X. C. Liu Z. H. Li G. H. Wang Q. S. (2014). Endosperm structure of white-belly and white-core rice grains shown by scanning electron microscopy. Plant Prod. Sci. 17, 285–290. 10.1626/pps.17.285

[ref57] Yamakawa H. Hakata M. (2010). Atlas of rice grain filling-related metabolism under high temperature: joint analysis of metabolome and transcriptome demonstrated inhibition of starch accumulation and induction of amino acid accumulation. Plant Cell Physiol. 51, 795–809. 10.1093/pcp/pcq034, PMID: 20304786 PMC2871029

[ref58] Yang S. X. Johnston N. Talideh E. Mitchell S. Jeffree C. Goodrich J.. (2008). The endosperm-specific *ZHOUPI* gene of *Arabidopsis thaliana* regulates endosperm breakdown and embryonic epidermal development. Development 135, 3501–3509. 10.1242/dev.026708, PMID: 18849529

[ref59] Yang X. Y. Lin Z. M. Liu Z. H. Ajim M. A. Bi J. G. Li G. H. (2013). Physicochemical and sensory properties of japonica rice varied with production areas in China. J. Integr. Agric. 12, 1748–1756. 10.1016/S2095-3119(13)60338-X

[ref60] Zeeman S. C. (2015). “Carbohydrate metabolism” in Biochemistry and molecular biology of plants. 2nd Edn. eds. Buchanan B. B. Gruissem W. Jones R. L. (Chichester: John Wiley & Sons, Ltd), 567–609.

[ref61] Zheng X. X. Li Q. Li C. S. An D. Xiao Q. Wang W. Q.. (2019). Intra-kernel reallocation of proteins in maize depends on VP1-mediated scutellum development and nutrient assimilation. Plant Cell 31, 2613–2635. 10.1105/tpc.19.00444, PMID: 31530735 PMC6881121

[ref62] Zhou S. R. Yin L. L. Xue H. W. (2013). Functional genomics based understanding of rice endosperm development. Curr. Opin. Plant Biol. 16, 236–246. 10.1016/j.pbi.2013.03.001, PMID: 23582455

[ref63] Zhu G. H. Ye N. H. Yang J. C. Peng X. X. Zhang J. H. (2011). Regulation of expression of starch synthesis genes by ethylene and ABA in relation to the development of rice inferior and superior spikelets. J. Exp. Bot. 62, 3907–3916. 10.1093/jxb/err088, PMID: 21459770 PMC3134347

